# Supplementary material for: An integrated hospital-district performance evaluation for communicable diseases in low-and middle-income countries: Evidence from a pilot in three sub-Saharan countries
Source: PLoS One. 2022 Mar 31;17(3):e0266225. doi: 10.1371/journal.pone.0266225 (PMC8970489; doi:10.1371/journal.pone.0266225)

S2 Fig. Trend and evaluated indicators related to the three CDs care pathways.

Fig. 3A. Trend and evaluation indicators related to tuberculosis care pathway.

IDPT06.1 Percentage of positive Xpert RR

Computational level: Hospital

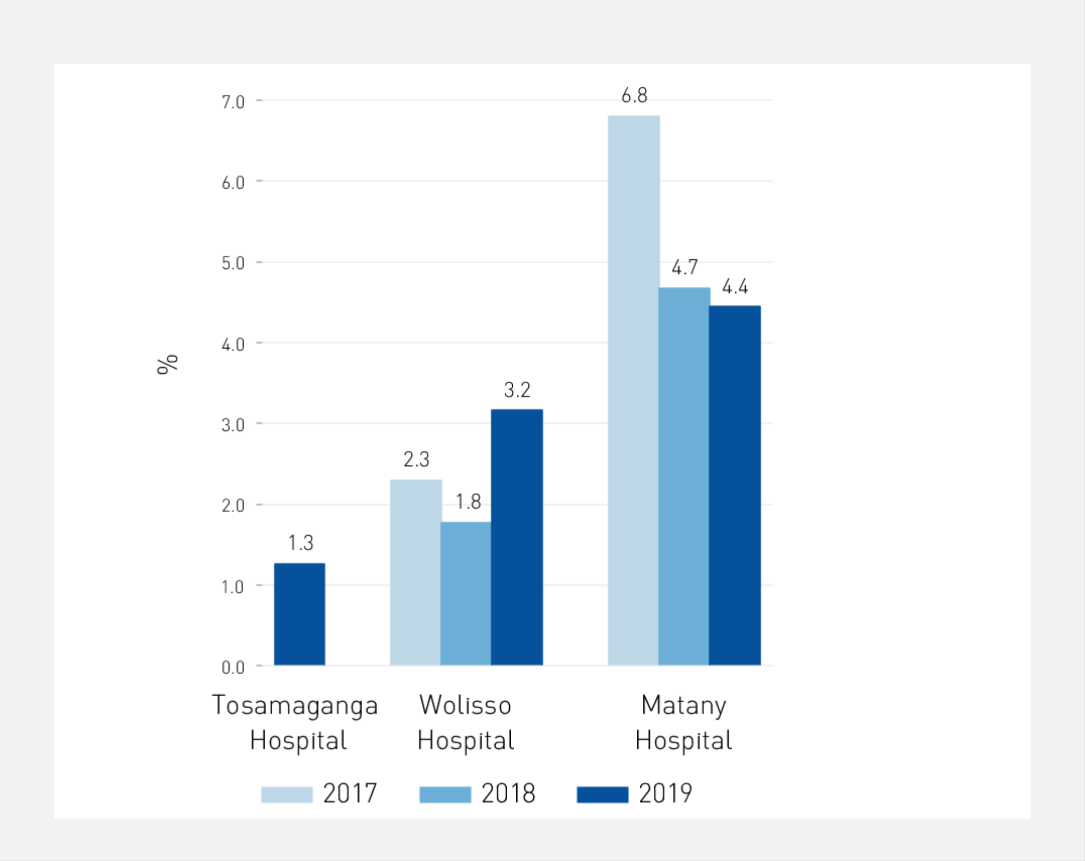

IDPT09 Percentage of cured patients

Computational level: District

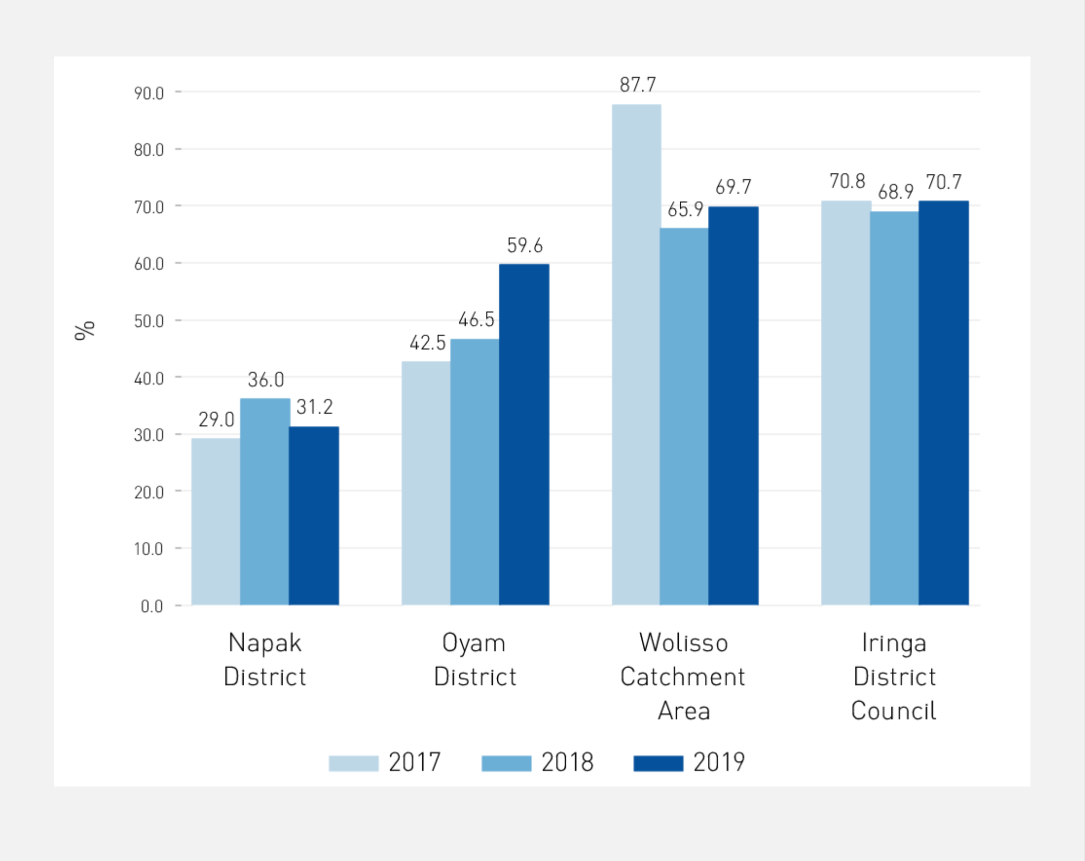

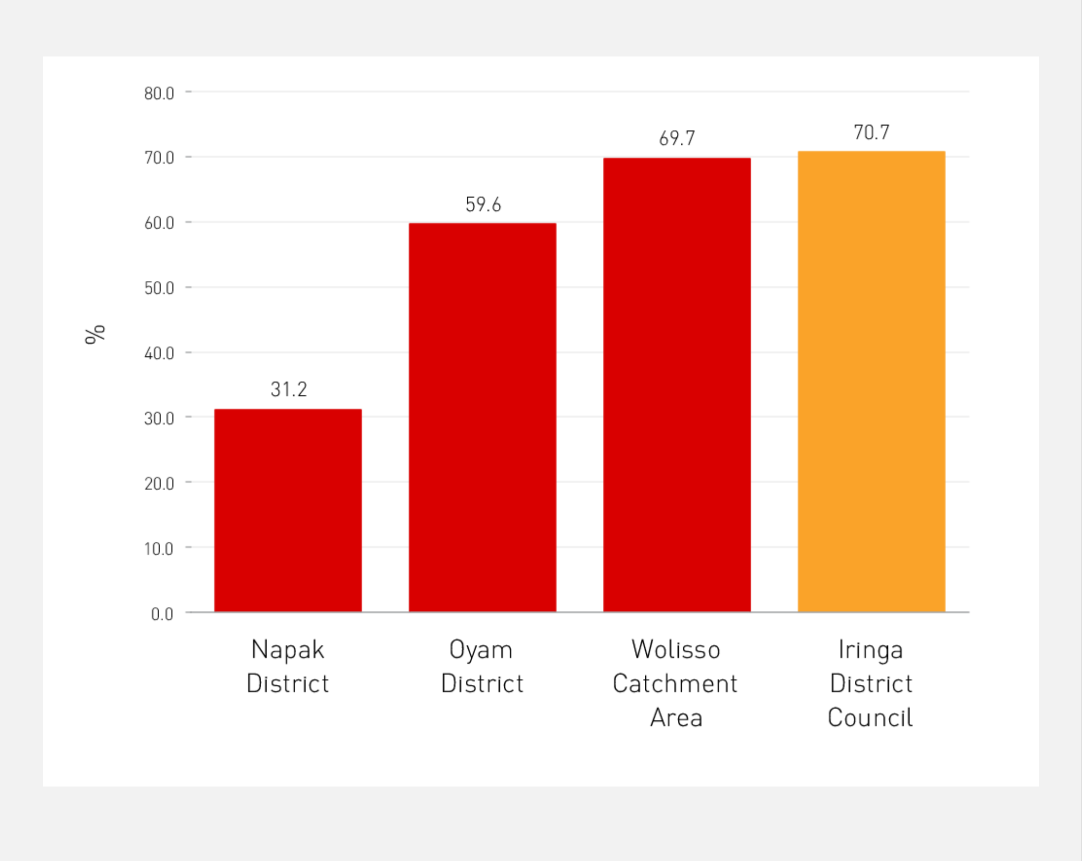

IDPT12 Percentage of interrupted treatments

Computational level: District

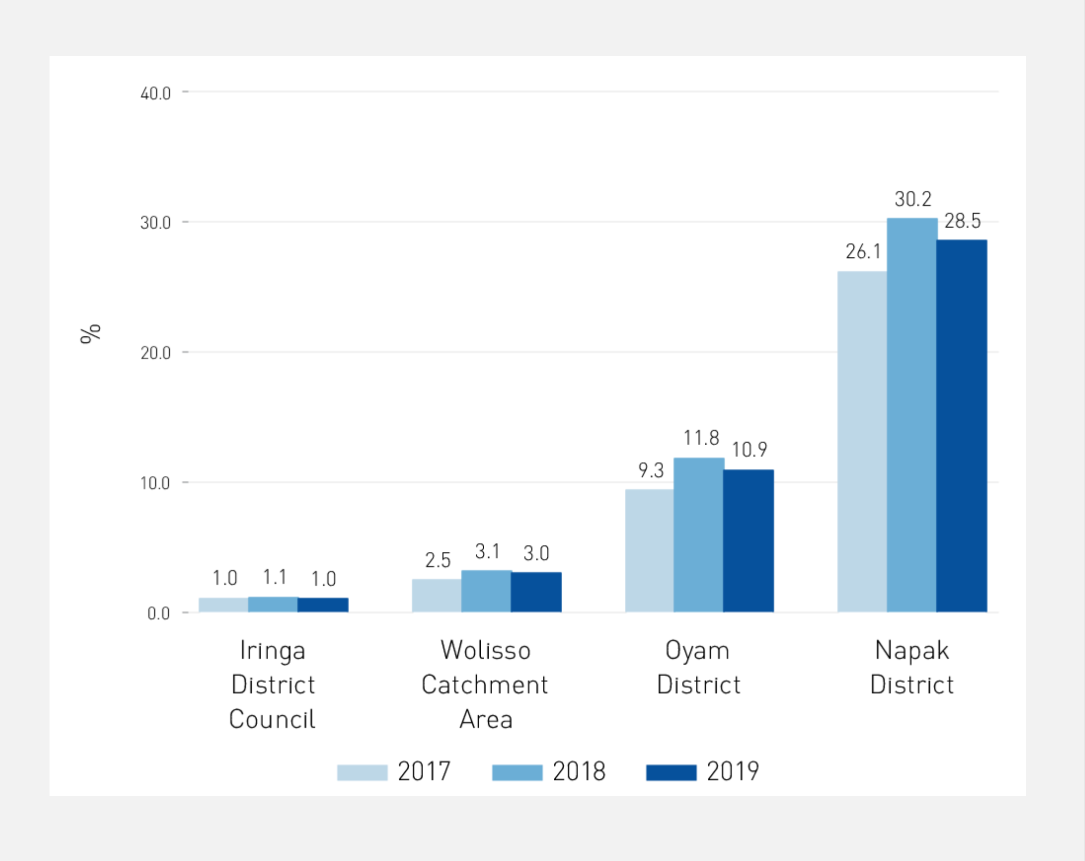

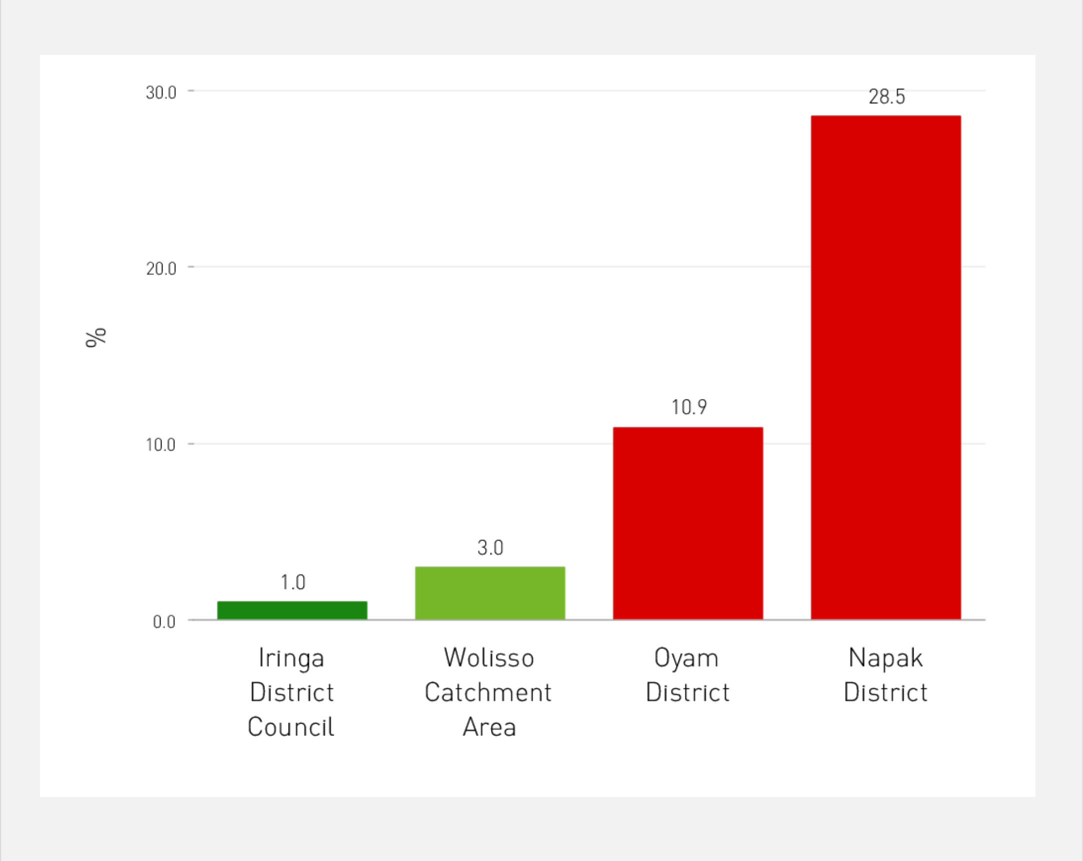

Fig. 3B. Trend indicator related to gastroenteritis care pathway.

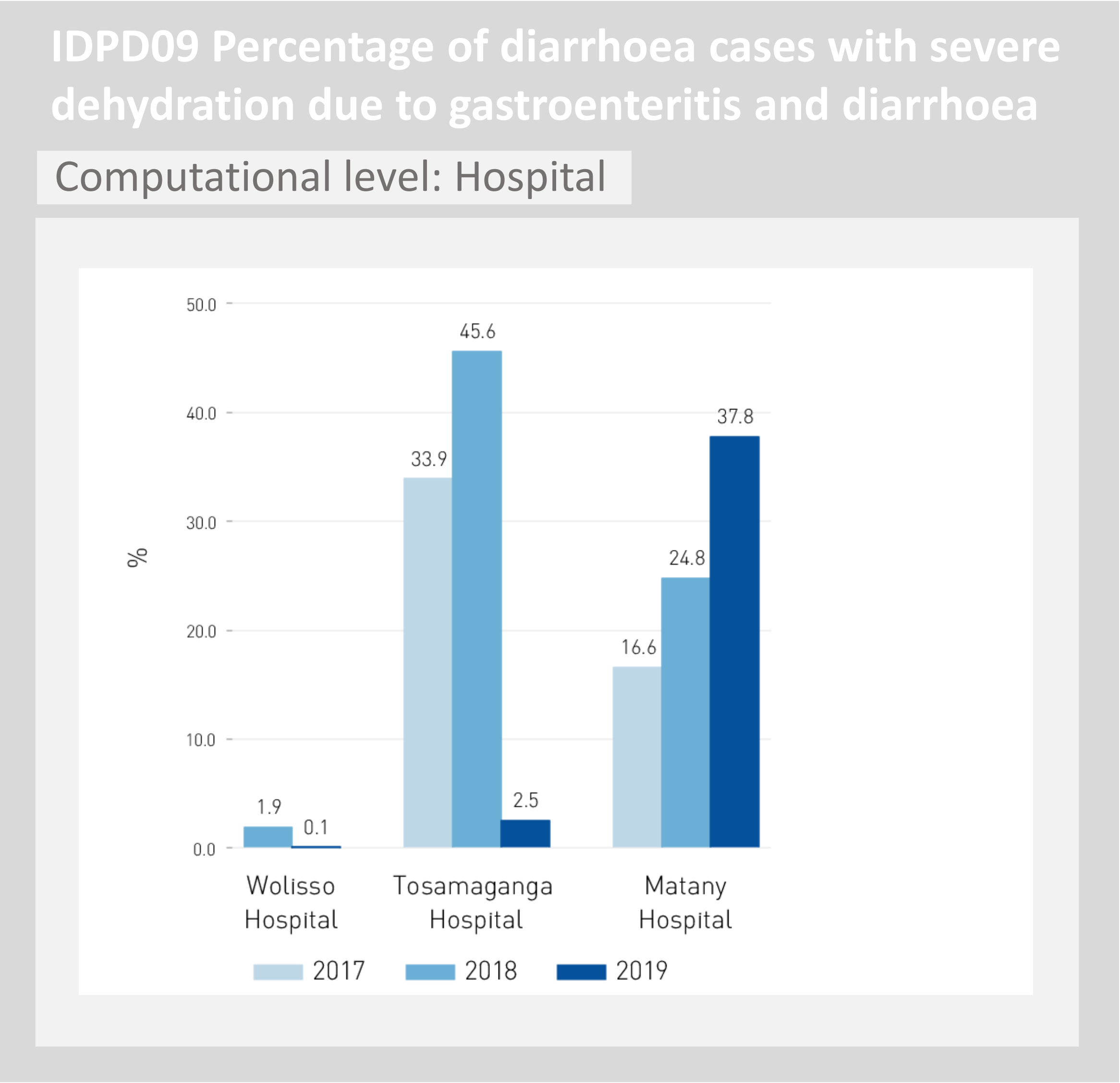

Fig. 3C. Trend indicators related to HIV/AIDS care pathway

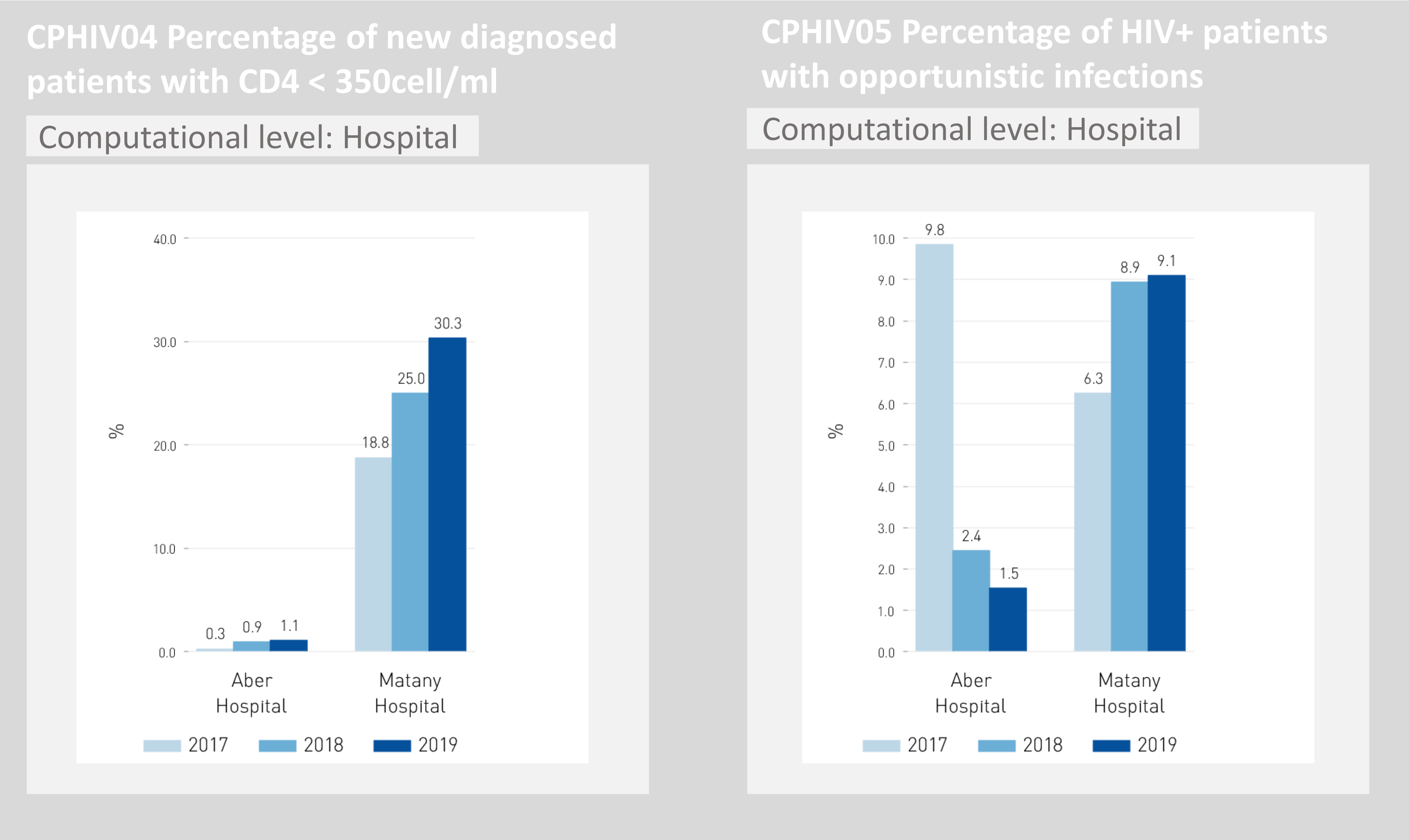

Supplement: S1 Fig — (PDF) [file pone.0266225.s001.pdf]
