# Supplementary material for: An integrated hospital-district performance evaluation for communicable diseases in low-and middle-income countries: Evidence from a pilot in three sub-Saharan countries
Source: PLoS One. 2022 Mar 31;17(3):e0266225. doi: 10.1371/journal.pone.0266225 (PMC8970489; doi:10.1371/journal.pone.0266225)
Supplement: S1 Table — (PDF) [file pone.0266225.s002.pdf]

S1 Table. List of the analysed hospitals and their relative health districts or catchment area.

Country	Region	Health District	Estimated population (Year 2019)	Reference Hospital	Hospital beds (2019)	Area (km2)	Population Density (citizens per km2)
Ethiopia	Oromia region	5 Woredas in Shoa-west Zone (Wolisso Town, Wolisso Rural, Ameya, Wonchi, Goro)	611 315	St. Luke - Wolisso Hospital	200	27 000	22.6
Tanzania	Iringa region	Iringa District Council	300 571	Tosamaganga District Designated Hospital	165	19 256	15.6
Uganda	Northern region	Napak District	156 989	St. Kizito - Matany Hospital	250	4978.4	31.5
Uganda	Northern region	Oyam District	432 050	Pope John XXIII - Aber Hospital	217	2190.8	197.2

With regard to Ethiopia, the information reported in the cell does not refer to an institutional health district, but to the catchment area covered by Wolisso Hospital.
