# Supplementary material for: An integrated hospital-district performance evaluation for communicable diseases in low-and middle-income countries: Evidence from a pilot in three sub-Saharan countries
Source: PLoS One. 2022 Mar 31;17(3):e0266225. doi: 10.1371/journal.pone.0266225 (PMC8970489; doi:10.1371/journal.pone.0266225)
Supplement: S1 File — (PDF) [file pone.0266225.s003.pdf]

S4 File. Link to the Report "Performance Evaluation System of hospital and health districts in Ethiopia, Uganda and Tanzania".

The report title "Performance Evaluation System of hospital and health districts in Ethiopia, Uganda and Tanzania" is available at the following link:

https://www.santannapisa.it/sites/default/files/report_cuamm19_webfv.pdf
