# Supplementary material for: An integrated hospital-district performance evaluation for communicable diseases in low-and middle-income countries: Evidence from a pilot in three sub-Saharan countries
Source: PLoS One. 2022 Mar 31;17(3):e0266225. doi: 10.1371/journal.pone.0266225 (PMC8970489; doi:10.1371/journal.pone.0266225)
Supplement: S2 File — (PDF) [file pone.0266225.s004.pdf]

S4 File. Data elements related to the indicators listed in Table 2.

Indicator code	Indicator name	Year	Computation level	Area	Numerator (description)	Denominator (description)	Numerator (value)	Denominator (value)	Indicator (Value)
B7.9	Vaccination coverage for rota virus	EEYY2011 (2019)	Residence	Wolisso Area	Number of children under one year of age who have received 2nd dose of Rota	Estimated number of infants aged less than 1 year	19.912	19.684	100,00
B7.9	Vaccination coverage for rota virus	EEYY2010 (2018)	Residence	Wolisso Area	Number of children under one year of age who have received 2nd dose of Rota	Estimated number of infants aged less than 1 year	12.906	19.280	66,94
B7.9	Vaccination coverage for rota virus	EEYY2009 (2017)	Residence	Wolisso Area	Number of children under one year of age who have received 2nd dose of Rota	Estimated number of infants aged less than 1 year	19.311	18.765	100,00
CPHIV02	Percentage of performed tests to pregnant women	EEYY2011 (2019)	Residence	Wolisso Area	Number of HIV performed tests to pregnant women followed at residence level	Total number of pregnant women with at least one ANC visit	13.775	19.940	69,08
CPHIV02	Percentage of performed tests to pregnant women	EEYY2010 (2018)	Residence	Wolisso Area	Number of HIV performed tests to pregnant women followed at residence level	Total number of pregnant women with at least one ANC visit	14.251	20.082	70,96
CPHIV02	Percentage of performed tests to pregnant women	EEYY2009 (2017)	Residence	Wolisso Area	Number of HIV performed tests to pregnant women followed at residence level	Total number of pregnant women with at least one ANC visit	12.136	20.726	58,55
CPHIV07	Percentage of new HIV+ linked to ART	EEYY2011 (2019)	Residence	Wolisso Area	Number of HIV+ starting ART (x100)	Number of new patients tested HIV+ in OPD and IPD	93	160	58,13
CPHIV07	Percentage of new HIV+ linked to ART	EEYY2010 (2018)	Residence	Wolisso Area	Number of HIV+ starting ART (x100)	Number of new patients tested HIV+ in OPD and IPD	213	394	54,06
CPHIV07	Percentage of new HIV+ linked to ART	EEYY2009 (2017)	Residence	Wolisso Area	Number of HIV+ starting ART (x100)	Number of new patients tested HIV+ in OPD and IPD	166	253	65,61
CPHIV08	Coverage rate of the therapy	EEYY2011 (2019)	Residence	Wolisso Area	Number of HIV+ patients currently on ART therapy (x100)	Number of HIV+ residents (estimated)	1.560	5.502	28,35
CPHIV08	Coverage rate of the therapy	EEYY2010 (2018)	Residence	Wolisso Area	Number of HIV+ patients currently on ART therapy (x100)	Number of HIV+ residents (estimated)	1.623	5.389	30,12
CPHIV08	Coverage rate of the therapy	EEYY2009 (2017)	Residence	Wolisso Area	Number of HIV+ patients currently on ART therapy (x100)	Number of HIV+ residents (estimated)	1.547	5.245	29,50
CPHIV10	Percentage of VL tests over the patients undergoing ART therapy	2019	Hospital	Wolisso Area	Number of patients undergoing VL tests (x100)	Number of patients currently on ART therapy	1241	1560	79,55
CPHIV10	Percentage of VL tests over the patients undergoing ART therapy	2018	Hospital	Wolisso Area	Number of patients undergoing VL tests (x100)	Number of patients currently on ART therapy	1089	1623	67,10
CPHIV10	Percentage of VL tests over the patients undergoing ART therapy	2017	Hospital	Wolisso Area	Number of patients undergoing VL tests (x100)	Number of patients currently on ART therapy	693	1547	44,80
CPHIV11	Percentage of patients undergoing ART therapy and tested with VL with suppression of viremia	2019	Hospital	Wolisso Area	Number of patients undergoing VL tests with viremia suppression (x100)	Number of patients currently on ART therapy and tested with VL within last 12 months	1.108	1241	89,28
CPHIV11	Percentage of patients undergoing ART therapy and tested with VL with suppression of viremia	2018	Hospital	Wolisso Area	Number of patients undergoing VL tests with viremia suppression (x100)	Number of patients currently on ART therapy and tested with VL within last 12 months	923	1089	84,76
CPHIV11	Percentage of patients undergoing ART therapy and tested with VL with suppression of viremia	2017	Hospital	Wolisso Area	Number of patients undergoing VL tests with viremia suppression (x100)	Number of patients currently on ART therapy and tested with VL within last 12 months	586	693	84,56
IDPD02	Average number of water sources by Hospital	2019	Hospital	Wolisso Area	Number of water taps	Total wards and outpatient rooms	52	44	1,18
IDPD02	Average number of water sources by Hospital	2018	Hospital	Wolisso Area	Number of water taps	Total wards and outpatient rooms	52	44	1,18
IDPD02	Average number of water sources by Hospital	2017	Hospital	Wolisso Area	Number of water taps	Total wards and outpatient rooms	52	44	1,18
IDPD04	Average number of toilets per bed in IPD	2019	Hospital	Wolisso Area	Number of toilets	Number of beds	32	200	0,16
IDPD04	Average number of toilets per bed in IPD	2018	Hospital	Wolisso Area	Number of toilets	Number of beds	32	200	0,16
IDPD04	Average number of toilets per bed in IPD	2017	Hospital	Wolisso Area	Number of toilets	Number of beds	32	200	0,16
IDPD05	Average number of toilets in OPD per number of rooms	2019	Hospital	Wolisso Area	Number of toilets in outpatient department (OPD)	Number of rooms in outpatient department (OPD)	4	17	0,24
IDPD05	Average number of toilets in OPD per number of rooms	2018	Hospital	Wolisso Area	Number of toilets in outpatient department (OPD)	Number of rooms in outpatient department (OPD)	4	17	0,24
IDPD05	Average number of toilets in OPD per number of rooms	2017	Hospital	Wolisso Area	Number of toilets in outpatient department (OPD)	Number of rooms in outpatient department (OPD)	4	17	0,24
IDPD10	Percentage of discharged patients for diarrhoea and gastroenteritis	2019	Hospital	Wolisso Area	Number of discharged patients for diarrhoea and gastroenteritis (x100)	Total number of discharged patients (adults and children)	760	14.892	5,10
IDPD10	Percentage of discharged patients for diarrhoea and gastroenteritis	2018	Hospital	Wolisso Area	Number of discharged patients for diarrhoea and gastroenteritis (x100)	Total number of discharged patients (adults and children)	779	15.086	5,16
IDPD10	Percentage of discharged patients for diarrhoea and gastroenteritis	2017	Hospital	Wolisso Area	Number of discharged patients for diarrhoea and gastroenteritis (x100)	Total number of discharged patients (adults and children)	950	14.990	6,34
IDPD14	Percentage of deaths with a diagnosis of gastroenteritis	2019	Hospital	Wolisso Area	Number of deaths diagnosed with gastroenteritis (patients aged < 5 years) (x10	Number of discharged patients with a diagnosis of gastroenteritis (patients aged < 5 years	5	653	0,77
IDPD14	Percentage of deaths with a diagnosis of gastroenteritis	2018	Hospital	Wolisso Area	Number of deaths diagnosed with gastroenteritis (patients aged < 5 years) (x10	Number of discharged patients with a diagnosis of gastroenteritis (patients aged < 5 years	2	666	0,30
IDPD14	Percentage of deaths with a diagnosis of gastroenteritis	2017	Hospital	Wolisso Area	Number of deaths diagnosed with gastroenteritis (patients aged < 5 years) (x10	Number of discharged patients with a diagnosis of gastroenteritis (patients aged < 5 years	8	822	0,97
IDPT02	Percentage of TB cases undergoing the HIV screening	EEYY2011 (2019)	Residence	Wolisso Area	Number of TB cases undergoing the HIV screening (x100)	Number of TB diagnosed patients	604	677	89,22
IDPT02	Percentage of TB cases undergoing the HIV screening	EEYY2010 (2018)	Residence	Wolisso Area	Number of TB cases undergoing the HIV screening (x100)	Number of TB diagnosed patients	327	392	83,42
IDPT02	Percentage of TB cases undergoing the HIV screening	EEYY2009 (2017)	Residence	Wolisso Area	Number of TB cases undergoing the HIV screening (x100)	Number of TB diagnosed patients	693	730	94,93
IDPT04	Percentage of confirmed TB cases on diagnosed cases	EEYY2011 (2019)	Residence	Wolisso Area	Number of positive PTB cases (bacteriologically confirmed) (x100)	Number of TB diagnosed patients	317	677	46,82
IDPT04	Percentage of confirmed TB cases on diagnosed cases	EEYY2010 (2018)	Residence	Wolisso Area	Number of positive PTB cases (bacteriologically confirmed) (x100)	Number of TB diagnosed patients	164	392	41,82
IDPT04	Percentage of confirmed TB cases on diagnosed cases	EEYY2009 (2017)	Residence	Wolisso Area	Number of positive PTB cases (bacteriologically confirmed) (x100)	Number of TB diagnosed patients	236	730	32,33
IDPT05	Percentage of confirmed PTB	EEYY2011 (2019)	Residence	Wolisso Area	Number of positive PTB cases (bacteriologically confirmed) (x100)	Number of PTB cases	317	526	60,27
IDPT05	Percentage of confirmed PTB	EEYY2010 (2018)	Residence	Wolisso Area	Number of positive PTB cases (bacteriologically confirmed) (x100)	Number of PTB cases	164	271	60,50
IDPT05	Percentage of confirmed PTB	EEYY2009 (2017)	Residence	Wolisso Area	Number of positive PTB cases (bacteriologically confirmed) (x100)	Number of PTB cases	236	391	60,36
IDPT06	Percentage of positive Xpert cases	2019	Hospital	Wolisso Area	Numer of positive Xpert cases (x100)	Number of Xpert cases	158	1.031	15,32
IDPT06	Percentage of positive Xpert cases	2018	Hospital	Wolisso Area	Numer of positive Xpert cases (x100)	Number of Xpert cases	281	1.814	15,49
IDPT06	Percentage of positive Xpert cases	2017	Hospital	Wolisso Area	Numer of positive Xpert cases (x100)	Number of Xpert cases	261	1.746	14,95
IDPT07	Percentage of treatments for extrapulmunary TB	EEYY2011 (2019)	Residence	Wolisso Area	Number of treatments "initiated" for extrapolmunary TB (x100)	Number of TB diagnoses	180	677	26,59
IDPT07	Percentage of treatments for extrapulmunary TB	EEYY2010 (2018)	Residence	Wolisso Area	Number of treatments "initiated" for extrapolmunary TB (x100)	Number of TB diagnoses	123	392	31,49
IDPT07	Percentage of treatments for extrapulmunary TB	EEYY2009 (2017)	Residence	Wolisso Area	Number of treatments "initiated" for extrapolmunary TB (x100)	Number of TB diagnoses	196	730	26,85
IDPT09	Percentage of cured patients	EEYY2011 (2019)	Residence	Wolisso Area	Number of cured patients (x100)	Number of PTB+ (bacteriologically confirmed)	221	317	69,72
IDPT09	Percentage of cured patients	EEYY2010 (2018)	Residence	Wolisso Area	Number of cured patients (x100)	Number of PTB+ (bacteriologically confirmed)	108	164	65,85
IDPT09	Percentage of cured patients	EEYY2009 (2017)	Residence	Wolisso Area	Number of cured patients (x100)	Number of PTB+ (bacteriologically confirmed)	207	236	87,71
IDPT10	Percentage of completed treatments	EEYY2011 (2019)	Residence	Wolisso Area	Number of completed treatments (x100)	Number of treated cases	470	506	92,89
IDPT10	Percentage of completed treatments	EEYY2010 (2018)	Residence	Wolisso Area	Number of completed treatments (x100)	Number of treated cases	234	257	91,05
IDPT10	Percentage of completed treatments	EEYY2009 (2017)	Residence	Wolisso Area	Number of completed treatments (x100)	Number of treated cases	521	570	91,40
IDPT12	Percentage of interrupted treatments	EEYY2011 (2019)	Residence	Wolisso Area	Number of interrupted treatments (x100)	Number of treated cases	15	506	2,96
IDPT12	Percentage of interrupted treatments	EEYY2010 (2018)	Residence	Wolisso Area	Number of interrupted treatments (x100)	Number of treated cases	8	257	3,11
IDPT12	Percentage of interrupted treatments	EEYY2009 (2017)	Residence	Wolisso Area	Number of interrupted treatments (x100)	Number of treated cases	14	570	2,46
B7.9	Vaccination coverage for rota virus	2019	Residence	Iringa DC	Number of children under one year of age who have received 2nd dose of Rota	Estimated number of infants aged less than 1 year	8381	9412	89,05
B7.9	Vaccination coverage for rota virus	2018	Residence	Iringa DC	Number of children under one year of age who have received 2nd dose of Rota	Estimated number of infants aged less than 1 year	8689	9286	93,57
B7.9	Vaccination coverage for rota virus	2017	Residence	Iringa DC	Number of children under one year of age who have received 2nd dose of Rota	Estimated number of infants aged less than 1 year	5938	9240	64,26
CPHIV02	Percentage of performed tests to pregnant women	2019	Residence	Iringa DC	Number of HIV performed tests to pregnant women followed at residence level	Total number of pregnant women with at least one ANC visit	9158	9646	94,94
CPHIV02	Percentage of performed tests to pregnant women	2018	Residence	Iringa DC	Number of HIV performed tests to pregnant women followed at residence level	Total number of pregnant women with at least one ANC visit	9183	9738	94,30
CPHIV02	Percentage of performed tests to pregnant women	2017	Residence	Iringa DC	Number of HIV performed tests to pregnant women followed at residence level	Total number of pregnant women with at least one ANC visit	9240	9870	93,62
CPHIV03	Percentage of HIV positive cases undergoing the TB screening	2019	Residence	Iringa DC	Number of HIV cases undergoing the TB screening (sputum, symptom question	Number of HIV+ cases	38288	43559	87,90
CPHIV03	Percentage of HIV positive cases undergoing the TB screening	2018	Residence	Iringa DC	Number of HIV cases undergoing the TB screening (sputum, symptom question	Number of HIV+ cases	26582	28877	92,05
CPHIV03	Percentage of HIV positive cases undergoing the TB screening	2017	Residence	Iringa DC	Number of HIV cases undergoing the TB screening (sputum, symptom question	Number of HIV+ cases	31289	32558	96,10
CPHIV07	Percentage of new HIV+ linked to ART	2019	Residence	Iringa DC	Number of HIV+ starting ART (x100)	Number of new patients tested HIV+ in OPD and IPD	1511	1511	100,00
CPHIV07	Percentage of new HIV+ linked to ART	2018	Residence	Iringa DC	Number of HIV+ starting ART (x100)	Number of new patients tested HIV+ in OPD and IPD	1360	1438	94,58
CPHIV07	Percentage of new HIV+ linked to ART	2017	Residence	Iringa DC	Number of HIV+ starting ART (x100)	Number of new patients tested HIV+ in OPD and IPD	1361	1361	100,00
CPHIV08	Coverage rate of the therapy	2019	Residence	Iringa DC	Number of HIV+ patients currently on ART therapy (x100)	Number of HIV+ residents (estimated)	11506	28554	40,30
CPHIV08	Coverage rate of the therapy	2018	Residence	Iringa DC	Number of HIV+ patients currently on ART therapy (x100)	Number of HIV+ residents (estimated)	9632	27912	34,51
CPHIV08	Coverage rate of the therapy	2017	Residence	Iringa DC	Number of HIV+ patients currently on ART therapy (x100)	Number of HIV+ residents (estimated)	8599	27215	31,60
CPHIV10	Percentage of VL tests over the patients undergoing ART therapy	2019	Hospital	Iringa DC	Number of patients undergoing VL tests (x100)	Number of patients currently on ART therapy	1452	1624	89,41
CPHIV10	Percentage of VL tests over the patients undergoing ART therapy	2018	Hospital	Iringa DC	Number of patients undergoing VL tests (x100)	Number of patients currently on ART therapy	1338	1428	93,70
CPHIV10	Percentage of VL tests over the patients undergoing ART therapy	2017	Hospital	Iringa DC	Number of patients undergoing VL tests (x100)	Number of patients currently on ART therapy	142	1385	10,25
CPHIV11	Percentage of patients undergoing ART therapy and tested with VL with suppression of viremia	2019	Hospital	Iringa DC	Number of patients undergoing VL tests with viremia suppression (x100)	Number of patients currently on ART therapy and tested with VL within last 12 months	979	1452	67,42
CPHIV11	Percentage of patients undergoing ART therapy and tested with VL with suppression of viremia	2018	Hospital	Iringa DC	Number of patients undergoing VL tests with viremia suppression (x100)	Number of patients currently on ART therapy and tested with VL within last 12 months	1001	1338	74,81
CPHIV11	Percentage of patients undergoing ART therapy and tested with VL with suppression of viremia	2017	Hospital	Iringa DC	Number of patients undergoing VL tests with viremia suppression (x100)	Number of patients currently on ART therapy and tested with VL within last 12 months	62	142	43,66
IDPD02	Average number of water sources by Hospital	2019	Hospital	Iringa DC	Number of water taps	Total wards and outpatient rooms	23	11	2,09
IDPD02	Average number of water sources by Hospital	2018	Hospital	Iringa DC	Number of water taps	Total wards and outpatient rooms	23	11	2,09
IDPD02	Average number of water sources by Hospital	2017	Hospital	Iringa DC	Number of water taps	Total wards and outpatient rooms	27	11	2,45
IDPD04	Average number of toilets per bed in IPD	2019	Hospital	Iringa DC	Number of toilets	Number of beds	18	165	0,11
IDPD04	Average number of toilets per bed in IPD	2018	Hospital	Iringa DC	Number of toilets	Number of beds	18	165	0,11

IDPD04	Average number of toilets per bed in IPD	2017	Hospital	Iringa DC	Number of toilets	Number of beds	18	165	0,11
IDPD05	Average number of toilets in OPD per number of rooms	2019	Hospital	Iringa DC	Number of toilets in outpatient department (OPD)	Number of rooms in outpatient department (OPD)	4	6	0,67
IDPD05	Average number of toilets in OPD per number of rooms	2018	Hospital	Iringa DC	Number of toilets in outpatient department (OPD)	Number of rooms in outpatient department (OPD)	4	6	0,67
IDPD05	Average number of toilets in OPD per number of rooms	2017	Hospital	Iringa DC	Number of toilets in outpatient department (OPD)	Number of rooms in outpatient department (OPD)	4	6	0,67
IDPD10	Percentage of discharged patients for diarrhoea and gastroenteritis	2019	Hospital	Iringa DC	Number of discharged patients for diarrhoea and gastroenteritis (x100)	Total number of discharged patients (adults and children)	121	6065	2,00
IDPD10	Percentage of discharged patients for diarrhoea and gastroenteritis	2018	Hospital	Iringa DC	Number of discharged patients for diarrhoea and gastroenteritis (x100)	Total number of discharged patients (adults and children)	118	6714	1,76
IDPD10	Percentage of discharged patients for diarrhoea and gastroenteritis	2017	Hospital	Iringa DC	Number of discharged patients for diarrhoea and gastroenteritis (x100)	Total number of discharged patients (adults and children)	107	6359	1,68
IDPD14	Percentage of deaths with a diagnosis of gastroenteritis	2019	Hospital	Iringa DC	Number of deaths diagnosed with gastroenteritis (patients aged < 5 years) (x10	Number of discharged patients with a diagnosis of gastroenteritis (patients aged < 5 years	2	101	1,98
IDPD14	Percentage of deaths with a diagnosis of gastroenteritis	2018	Hospital	Iringa DC	Number of deaths diagnosed with gastroenteritis (patients aged < 5 years) (x10	Number of discharged patients with a diagnosis of gastroenteritis (patients aged < 5 years	2	85	2,35
IDPD14	Percentage of deaths with a diagnosis of gastroenteritis	2017	Hospital	Iringa DC	Number of deaths diagnosed with gastroenteritis (patients aged < 5 years) (x10	Number of discharged patients with a diagnosis of gastroenteritis (patients aged < 5 years	2	68	2,94
IDPT01	Percentage of treatments with isoniazide (IPT)	2019	Residence	Iringa DC	Number of treatments with isoniazide (IPT) (x100)	Total number of eligible treatments	5107	38288	13,34
IDPT01	Percentage of treatments with isoniazide (IPT)	2018	Residence	Iringa DC	Number of treatments with isoniazide (IPT) (x100)	Total number of eligible treatments	1953	26582	7,35
IDPT01	Percentage of treatments with isoniazide (IPT)	2017	Residence	Iringa DC	Number of treatments with isoniazide (IPT) (x100)	Total number of eligible treatments	151	31289	0,48
IDPT02	Percentage of TB cases undergoing the HIV screening	2019	Residence	Iringa DC	Number of TB cases undergoing the HIV screening (x100)	Number of TB diagnosed patients	387	391	98,98
IDPT02	Percentage of TB cases undergoing the HIV screening	2018	Residence	Iringa DC	Number of TB cases undergoing the HIV screening (x100)	Number of TB diagnosed patients	335	336	99,70
IDPT02	Percentage of TB cases undergoing the HIV screening	2017	Residence	Iringa DC	Number of TB cases undergoing the HIV screening (x100)	Number of TB diagnosed patients	404	413	97,82
IDPT04	Percentage of confirmed TB cases on diagnosed cases	2019	Residence	Iringa DC	Number of positive PTB cases (bacteriologically confirmed) (x100)	Number of TB diagnosed patients	133	397	33,50
IDPT04	Percentage of confirmed TB cases on diagnosed cases	2018	Residence	Iringa DC	Number of positive PTB cases (bacteriologically confirmed) (x100)	Number of TB diagnosed patients	135	338	39,94
IDPT04	Percentage of confirmed TB cases on diagnosed cases	2017	Residence	Iringa DC	Number of positive PTB cases (bacteriologically confirmed) (x100)	Number of TB diagnosed patients	171	417	41,01
IDPT05	Percentage of confirmed PTB	2019	Residence	Iringa DC	Number of positive PTB cases (bacteriologically confirmed) (x100)	Number of PTB cases	133	334	39,82
IDPT05	Percentage of confirmed PTB	2018	Residence	Iringa DC	Number of positive PTB cases (bacteriologically confirmed) (x100)	Number of PTB cases	135	284	47,54
IDPT05	Percentage of confirmed PTB	2017	Residence	Iringa DC	Number of positive PTB cases (bacteriologically confirmed) (x100)	Number of PTB cases	171	361	47,37
IDPT06	Percentage of positive Xpert cases	2019	Hospital	Iringa DC	Numer of positive Xpert cases (x100)	Number of Xpert cases	79	1240	6,37
IDPT06	Percentage of positive Xpert cases	2018	Hospital	Iringa DC	Numer of positive Xpert cases (x100)	Number of Xpert cases	51	733	6,96
IDPT07	Percentage of treatments for extrapulmunary TB	2019	Residence	Iringa DC	Number of treatments "initiated" for extrapolmunary TB (x100)	Number of TB diagnoses	63	391	16,11
IDPT07	Percentage of treatments for extrapulmunary TB	2018	Residence	Iringa DC	Number of treatments "initiated" for extrapolmunary TB (x100)	Number of TB diagnoses	54	336	16,07
IDPT07	Percentage of treatments for extrapulmunary TB	2017	Residence	Iringa DC	Number of treatments "initiated" for extrapolmunary TB (x100)	Number of TB diagnoses	56	413	13,56
IDPT09	Percentage of cured patients	2019	Residence	Iringa DC	Number of cured patients (x100)	Number of PTB+ (bacteriologically confirmed)	117	133	87,97
IDPT09	Percentage of cured patients	2018	Residence	Iringa DC	Number of cured patients (x100)	Number of PTB+ (bacteriologically confirmed)	92	135	68,15
IDPT09	Percentage of cured patients	2017	Residence	Iringa DC	Number of cured patients (x100)	Number of PTB+ (bacteriologically confirmed)	121	171	70,76
IDPT10	Percentage of completed treatments	2019	Residence	Iringa DC	Number of completed treatments (x100)	Number of treated cases	355	391	90,79
IDPT10	Percentage of completed treatments	2018	Residence	Iringa DC	Number of completed treatments (x100)	Number of treated cases	331	335	98,81
IDPT10	Percentage of completed treatments	2017	Residence	Iringa DC	Number of completed treatments (x100)	Number of treated cases	351	413	84,99
IDPT12	Percentage of interrupted treatments	2019	Residence	Iringa DC	Number of interrupted treatments (x100)	Number of treated cases	3	391	0,77
IDPT12	Percentage of interrupted treatments	2018	Residence	Iringa DC	Number of interrupted treatments (x100)	Number of treated cases	3	335	0,90
IDPT12	Percentage of interrupted treatments	2017	Residence	Iringa DC	Number of interrupted treatments (x100)	Number of treated cases	3	413	0,73
B7.9	Vaccination coverage for rota virus	2019	Residence	Napak District	Number of children under one year of age who have received 2nd dose of Rota	Estimated number of infants aged less than 1 year	14013	6751	100,0
B7.9	Vaccination coverage for rota virus	2018	Residence	Napak District	Number of children under one year of age who have received 2nd dose of Rota	Estimated number of infants aged less than 1 year	999	6554	15,2
B7.9	Vaccination coverage for rota virus	2017	Residence	Napak District	Number of children under one year of age who have received 2nd dose of Rota	Estimated number of infants aged less than 1 year	346	6376	5,4
CPHIV02	Percentage of performed tests to pregnant women	2019	Residence	Napak District	Number of HIV performed tests to pregnant women followed at residence level	Total number of pregnant women with at least one ANC visit	6697	7641	87,6
CPHIV02	Percentage of performed tests to pregnant women	2018	Residence	Napak District	Number of HIV performed tests to pregnant women followed at residence level	Total number of pregnant women with at least one ANC visit	6083	6802	89,4
CPHIV02	Percentage of performed tests to pregnant women	2017	Residence	Napak District	Number of HIV performed tests to pregnant women followed at residence level	Total number of pregnant women with at least one ANC visit	6627	6777	97,8
CPHIV03	Percentage of HIV positive cases undergoing the TB screening	2019	Residence	Napak District	Number of HIV cases undergoing the TB screening (sputum, symptom question	Number of HIV+ cases	4243	4353	97,5
CPHIV03	Percentage of HIV positive cases undergoing the TB screening	2018	Residence	Napak District	Number of HIV cases undergoing the TB screening (sputum, symptom question	Number of HIV+ cases	2279	3222	70,7
CPHIV03	Percentage of HIV positive cases undergoing the TB screening	2017	Residence	Napak District	Number of HIV cases undergoing the TB screening (sputum, symptom question	Number of HIV+ cases	3502	3485	100,5
CPHIV07	Percentage of new HIV+ linked to ART	2019	Residence	Napak District	Number of HIV+ starting ART (x100)	Number of new patients tested HIV+ in OPD and IPD	269	309	87,1
CPHIV07	Percentage of new HIV+ linked to ART	2018	Residence	Napak District	Number of HIV+ starting ART (x100)	Number of new patients tested HIV+ in OPD and IPD	132	155	85,2
CPHIV07	Percentage of new HIV+ linked to ART	2017	Residence	Napak District	Number of HIV+ starting ART (x100)	Number of new patients tested HIV+ in OPD and IPD	188	209	90,0
CPHIV08	Coverage rate of the therapy	2019	Residence	Napak District	Number of HIV+ patients currently on ART therapy (x100)	Number of HIV+ residents (estimated)	9949	8320	119,6
CPHIV08	Coverage rate of the therapy	2018	Residence	Napak District	Number of HIV+ patients currently on ART therapy (x100)	Number of HIV+ residents (estimated)	9496	8078	117,6
CPHIV08	Coverage rate of the therapy	2017	Residence	Napak District	Number of HIV+ patients currently on ART therapy (x100)	Number of HIV+ residents (estimated)	8964	7859	114,1
CPHIV10	Percentage of VL tests over the patients undergoing ART therapy	2019	Hospital	Napak District	Number of patients undergoing VL tests (x100)	Number of patients currently on ART therapy	444	5643	7,9
CPHIV10	Percentage of VL tests over the patients undergoing ART therapy	2018	Hospital	Napak District	Number of patients undergoing VL tests (x100)	Number of patients currently on ART therapy	425	5510	7,7
CPHIV10	Percentage of VL tests over the patients undergoing ART therapy	2017	Hospital	Napak District	Number of patients undergoing VL tests (x100)	Number of patients currently on ART therapy	433	5233	8,3
CPHIV11	Percentage of patients undergoing ART therapy and tested with VL with suppression of viremia	2019	Hospital	Napak District	Number of patients undergoing VL tests with viremia suppression (x100)	Number of patients currently on ART therapy and tested with VL within last 12 months	358	444	80,6
CPHIV11	Percentage of patients undergoing ART therapy and tested with VL with suppression of viremia	2018	Hospital	Napak District	Number of patients undergoing VL tests with viremia suppression (x100)	Number of patients currently on ART therapy and tested with VL within last 12 months	305	425	71,8
CPHIV11	Percentage of patients undergoing ART therapy and tested with VL with suppression of viremia	2017	Hospital	Napak District	Number of patients undergoing VL tests with viremia suppression (x100)	Number of patients currently on ART therapy and tested with VL within last 12 months	294	433	67,9
IDPD02	Average number of water sources by Hospital	2019	Hospital	Napak District	Number of water taps	Total wards and outpatient rooms	49	116	0,4
IDPD02	Average number of water sources by Hospital	2018	Hospital	Napak District	Number of water taps	Total wards and outpatient rooms	46	115	0,4
IDPD02	Average number of water sources by Hospital	2017	Hospital	Napak District	Number of water taps	Total wards and outpatient rooms	32	115	0,3
IDPD04	Average number of toilets per bed in IPD	2020	Hospital	Napak District	Number of toilets	Number of beds	62	250	0,2
IDPD04	Average number of toilets per bed in IPD	2018	Hospital	Napak District	Number of toilets	Number of beds	60	250	0,2
IDPD04	Average number of toilets per bed in IPD	2017	Hospital	Napak District	Number of toilets	Number of beds	56	250	0,2
IDPD05	Average number of toilets in OPD per number of rooms	2019	Hospital	Napak District	Number of toilets in outpatient department (OPD)	Number of rooms in outpatient department (OPD)	4	13	0,3
IDPD05	Average number of toilets in OPD per number of rooms	2018	Hospital	Napak District	Number of toilets in outpatient department (OPD)	Number of rooms in outpatient department (OPD)	4	13	0,3
IDPD05	Average number of toilets in OPD per number of rooms	2017	Hospital	Napak District	Number of toilets in outpatient department (OPD)	Number of rooms in outpatient department (OPD)	4	13	0,3
IDPD10	Percentage of discharged patients for diarrhoea and gastroenteritis	2019	Hospital	Napak District	Number of discharged patients for diarrhoea and gastroenteritis (x100)	Total number of discharged patients (adults and children)	744	7591	9,8
IDPD10	Percentage of discharged patients for diarrhoea and gastroenteritis	2018	Hospital	Napak District	Number of discharged patients for diarrhoea and gastroenteritis (x100)	Total number of discharged patients (adults and children)	585	8006	7,3
IDPD10	Percentage of discharged patients for diarrhoea and gastroenteritis	2017	Hospital	Napak District	Number of discharged patients for diarrhoea and gastroenteritis (x100)	Total number of discharged patients (adults and children)	603	8210	7,3
IDPD12	Average number of ORS packages delivered per patient with diarrhoea (<5years)	2019	Residence	Napak District	Number of ORS packages delivered (Hospital + Health Centers)	Total number of diarrhoea cases (<5 years)	12521	5223	2,4
IDPD12	Average number of ORS packages delivered per patient with diarrhoea (<5years)	2018	Residence	Napak District	Number of ORS packages delivered (Hospital + Health Centers)	Total number of diarrhoea cases (<5 years)	11302	4660	2,4
IDPD12	Average number of ORS packages delivered per patient with diarrhoea (<5years)	2017	Residence	Napak District	Number of ORS packages delivered (Hospital + Health Centers)	Total number of diarrhoea cases (<5 years)	12851	5144	2,5
IDPD13	Average number of Zinc Tablets doses delivered per patient with diarrhoea (<5years)	2019	Residence	Napak District	Number of Zinc Tablets doses delivered (Hospital + Health Centers)	Total number of diarrhoea cases (<5 years)	12521	5223	2,4
IDPD13	Average number of Zinc Tablets doses delivered per patient with diarrhoea (<5years)	2018	Residence	Napak District	Number of Zinc Tablets doses delivered (Hospital + Health Centers)	Total number of diarrhoea cases (<5 years)	11302	4660	2,4
IDPD13	Average number of Zinc Tablets doses delivered per patient with diarrhoea (<5years)	2017	Residence	Napak District	Number of Zinc Tablets doses delivered (Hospital + Health Centers)	Total number of diarrhoea cases (<5 years)	12851	5144	2,5
IDPD14	Percentage of deaths with a diagnosis of gastroenteritis	2019	Hospital	Napak District	Number of deaths diagnosed with gastroenteritis (patients aged < 5 years) (x10	Number of discharged patients with a diagnosis of gastroenteritis (patients aged < 5 years	6	535	1,1
IDPD14	Percentage of deaths with a diagnosis of gastroenteritis	2018	Hospital	Napak District	Number of deaths diagnosed with gastroenteritis (patients aged < 5 years) (x10	Number of discharged patients with a diagnosis of gastroenteritis (patients aged < 5 years	2	395	0,5
IDPD14	Percentage of deaths with a diagnosis of gastroenteritis	2017	Hospital	Napak District	Number of deaths diagnosed with gastroenteritis (patients aged < 5 years) (x10	Number of discharged patients with a diagnosis of gastroenteritis (patients aged < 5 years	4	414	1,0
IDPT01	Percentage of treatments with isoniazide (IPT)	2019	Residence	Napak District	Number of treatments with isoniazide (IPT) (x100)	Total number of eligible treatments	68	413	16,5
IDPT01	Percentage of treatments with isoniazide (IPT)	2018	Residence	Napak District	Number of treatments with isoniazide (IPT) (x100)	Total number of eligible treatments	23	324	7,1
IDPT01	Percentage of treatments with isoniazide (IPT)	2017	Residence	Napak District	Number of treatments with isoniazide (IPT) (x100)	Total number of eligible treatments	1	50	2,0
IDPT02	Percentage of TB cases undergoing the HIV screening	2019	Residence	Napak District	Number of TB cases undergoing the HIV screening (x100)	Number of TB diagnosed patients	747	838	89,1
IDPT02	Percentage of TB cases undergoing the HIV screening	2018	Residence	Napak District	Number of TB cases undergoing the HIV screening (x100)	Number of TB diagnosed patients	746	798	93,5
IDPT02	Percentage of TB cases undergoing the HIV screening	2017	Residence	Napak District	Number of TB cases undergoing the HIV screening (x100)	Number of TB diagnosed patients	312	327	95,4
IDPT04	Percentage of confirmed TB cases on diagnosed cases	2019	Residence	Napak District	Number of positive PTB cases (bacteriologically confirmed) (x100)	Number of TB diagnosed patients	361	728	49,6
IDPT04	Percentage of confirmed TB cases on diagnosed cases	2018	Residence	Napak District	Number of positive PTB cases (bacteriologically confirmed) (x100)	Number of TB diagnosed patients	304	696	43,7
IDPT04	Percentage of confirmed TB cases on diagnosed cases	2017	Residence	Napak District	Number of positive PTB cases (bacteriologically confirmed) (x100)	Number of TB diagnosed patients	181	294	61,6

IDPT05	Percentage of confirmed PTB	2019	Residence	Napak District	Number of positive PTB cases (bacteriologically confirmed) (x100)	Number of PTB cases	361	584	61,8
IDPT05	Percentage of confirmed PTB	2018	Residence	Napak District	Number of positive PTB cases (bacteriologically confirmed) (x100)	Number of PTB cases	304	455	66,8
IDPT05	Percentage of confirmed PTB	2017	Residence	Napak District	Number of positive PTB cases (bacteriologically confirmed) (x100)	Number of PTB cases	181	212	85,4
IDPT06	Percentage of positive Xpert cases	2019	Hospital	Napak District	Numer of positive Xpert cases (x100)	Number of Xpert cases	360	3750	9,6
IDPT06	Percentage of positive Xpert cases	2018	Hospital	Napak District	Numer of positive Xpert cases (x100)	Number of Xpert cases	385	2966	13,0
IDPT06	Percentage of positive Xpert cases	2017	Hospital	Napak District	Numer of positive Xpert cases (x100)	Number of Xpert cases	338	1399	24,2
IDPT07	Percentage of treatments for extrapulmunary TB	2019	Residence	Napak District	Number of treatments "initiated" for extrapolmunary TB (x100)	Number of TB diagnoses	24	728	3,3
IDPT07	Percentage of treatments for extrapulmunary TB	2018	Residence	Napak District	Number of treatments "initiated" for extrapolmunary TB (x100)	Number of TB diagnoses	6	696	0,9
IDPT07	Percentage of treatments for extrapulmunary TB	2017	Residence	Napak District	Number of treatments "initiated" for extrapolmunary TB (x100)	Number of TB diagnoses	13	294	4,4
IDPT09	Percentage of cured patients	2019	Residence	Napak District	Number of cured patients (x100)	Number of PTB+ (bacteriologically confirmed)	129	367	35,1
IDPT09	Percentage of cured patients	2018	Residence	Napak District	Number of cured patients (x100)	Number of PTB+ (bacteriologically confirmed)	150	380	39,5
IDPT09	Percentage of cured patients	2017	Residence	Napak District	Number of cured patients (x100)	Number of PTB+ (bacteriologically confirmed)	46	181	25,4
IDPT10	Percentage of treatment success	2019	Residence	Napak District	Number of completed treatments (x100)	Number of treated cases	431	787	54,8
IDPT10	Percentage of treatment success	2018	Residence	Napak District	Number of completed treatments (x100)	Number of treated cases	306	612	50,0
IDPT10	Percentage of treatment success	2017	Residence	Napak District	Number of completed treatments (x100)	Number of treated cases	137	234	58,5
IDPT12	Percentage of interrupted treatments	2019	Residence	Napak District	Number of interrupted treatments (x100)	Number of treated cases	279	787	35,5
IDPT12	Percentage of interrupted treatments	2018	Residence	Napak District	Number of interrupted treatments (x100)	Number of treated cases	135	612	22,1
IDPT12	Percentage of interrupted treatments	2017	Residence	Napak District	Number of interrupted treatments (x100)	Number of treated cases	54	234	23,1
B7.9	Vaccination coverage for rota virus	2019	Residence	Oyam District	Number of children under one year of age who have received 2nd dose of Rota	Estimated number of infants aged less than 1 year	12956	18578	69,74
B7.9	Vaccination coverage for rota virus	2018	Residence	Oyam District	Number of children under one year of age who have received 2nd dose of Rota	Estimated number of infants aged less than 1 year	8	18082	0,04
B7.9	Vaccination coverage for rota virus	2017	Residence	Oyam District	Number of children under one year of age who have received 2nd dose of Rota	Estimated number of infants aged less than 1 year	11	17591	0,06
CPHIV02	Percentage of performed tests to pregnant women	2019	Residence	Oyam District	Number of HIV performed tests to pregnant women followed at residence level	Total number of pregnant women with at least one ANC visit	17570	21122	83,18
CPHIV02	Percentage of performed tests to pregnant women	2018	Residence	Oyam District	Number of HIV performed tests to pregnant women followed at residence level	Total number of pregnant women with at least one ANC visit	16850	19068	88,37
CPHIV02	Percentage of performed tests to pregnant women	2017	Residence	Oyam District	Number of HIV performed tests to pregnant women followed at residence level	Total number of pregnant women with at least one ANC visit	16507	19399	85,09
CPHIV03	Percentage of HIV positive cases undergoing the TB screening	2019	Residence	Oyam District	Number of HIV cases undergoing the TB screening (sputum, symptom question	Number of HIV+ cases	44627	48172	92,64
CPHIV03	Percentage of HIV positive cases undergoing the TB screening	2018	Residence	Oyam District	Number of HIV cases undergoing the TB screening (sputum, symptom question	Number of HIV+ cases	53420	54418	98,17
CPHIV03	Percentage of HIV positive cases undergoing the TB screening	2017	Residence	Oyam District	Number of HIV cases undergoing the TB screening (sputum, symptom question	Number of HIV+ cases	39386	42299	93,11
CPHIV07	Percentage of new HIV+ linked to ART	2019	Residence	Oyam District	Number of HIV+ starting ART (x100)	Number of new patients tested HIV+ in OPD and IPD	1989	2164	91,91
CPHIV07	Percentage of new HIV+ linked to ART	2018	Residence	Oyam District	Number of HIV+ starting ART (x100)	Number of new patients tested HIV+ in OPD and IPD	1912	2291	83,46
CPHIV07	Percentage of new HIV+ linked to ART	2017	Residence	Oyam District	Number of HIV+ starting ART (x100)	Number of new patients tested HIV+ in OPD and IPD	2287	2547	89,79
CPHIV08	Coverage rate of the therapy	2019	Residence	Oyam District	Number of HIV+ patients currently on ART therapy (x100)	Number of HIV+ residents (estimated)	12625	30244	41,74
CPHIV08	Coverage rate of the therapy	2018	Residence	Oyam District	Number of HIV+ patients currently on ART therapy (x100)	Number of HIV+ residents (estimated)	13372	29435	45,43
CPHIV08	Coverage rate of the therapy	2017	Residence	Oyam District	Number of HIV+ patients currently on ART therapy (x100)	Number of HIV+ residents (estimated)	10963	28637	38,28
CPHIV10	Percentage of VL tests over the patients undergoing ART therapy	2019	Hospital	Oyam District	Number of patients undergoing VL tests (x100)	Number of patients currently on ART therapy	1841	4850	38
CPHIV10	Percentage of VL tests over the patients undergoing ART therapy	2018	Hospital	Oyam District	Number of patients undergoing VL tests (x100)	Number of patients currently on ART therapy	402	5605	7
CPHIV10	Percentage of VL tests over the patients undergoing ART therapy	2017	Hospital	Oyam District	Number of patients undergoing VL tests (x100)	Number of patients currently on ART therapy	224	4215	5
CPHIV11	Percentage of patients undergoing ART therapy and tested with VL with suppression of viremia	2019	Hospital	Oyam District	Number of patients undergoing VL tests with viremia suppression (x100)	Number of patients currently on ART therapy and tested with VL within last 12 months	25	1841	1,21
CPHIV11	Percentage of patients undergoing ART therapy and tested with VL with suppression of viremia	2018	Hospital	Oyam District	Number of patients undergoing VL tests with viremia suppression (x100)	Number of patients currently on ART therapy and tested with VL within last 12 months	16	402	0,57
CPHIV11	Percentage of patients undergoing ART therapy and tested with VL with suppression of viremia	2017	Hospital	Oyam District	Number of patients undergoing VL tests with viremia suppression (x100)	Number of patients currently on ART therapy and tested with VL within last 12 months	31	224	1,51
IDPD02	Average number of water sources by Hospital	2019	Hospital	Oyam District	Number of water taps	Total wards and outpatient rooms	20	24	0,83
IDPD02	Average number of water sources by Hospital	2018	Hospital	Oyam District	Number of water taps	Total wards and outpatient rooms	15	20	0,75
IDPD02	Average number of water sources by Hospital	2017	Hospital	Oyam District	Number of water taps	Total wards and outpatient rooms	15	20	0,75
IDPD04	Average number of toilets per bed in IPD	2019	Hospital	Oyam District	Number of toilets	Number of beds	28	217	0,13
IDPD04	Average number of toilets per bed in IPD	2018	Hospital	Oyam District	Number of toilets	Number of beds	26	179	0,15
IDPD04	Average number of toilets per bed in IPD	2017	Hospital	Oyam District	Number of toilets	Number of beds	26	178	0,15
IDPD05	Average number of toilets in OPD per number of rooms	2019	Hospital	Oyam District	Number of toilets in outpatient department (OPD)	Number of rooms in outpatient department (OPD)	7	5	1,40
IDPD05	Average number of toilets in OPD per number of rooms	2018	Hospital	Oyam District	Number of toilets in outpatient department (OPD)	Number of rooms in outpatient department (OPD)	7	5	1,40
IDPD05	Average number of toilets in OPD per number of rooms	2017	Hospital	Oyam District	Number of toilets in outpatient department (OPD)	Number of rooms in outpatient department (OPD)	7	5	1,40
IDPD10	Percentage of discharged patients for diarrhoea and gastroenteritis	2019	Hospital	Oyam District	Number of discharged patients for diarrhoea and gastroenteritis (x100)	Total number of discharged patients (adults and children)	191	9771	1,95
IDPD10	Percentage of discharged patients for diarrhoea and gastroenteritis	2018	Hospital	Oyam District	Number of discharged patients for diarrhoea and gastroenteritis (x100)	Total number of discharged patients (adults and children)	261	8442	3,09
IDPD10	Percentage of discharged patients for diarrhoea and gastroenteritis	2017	Hospital	Oyam District	Number of discharged patients for diarrhoea and gastroenteritis (x100)	Total number of discharged patients (adults and children)	205	12252	1,67
IDPD12	Average number of ORS packages delivered per patient with diarrhoea (<5years)	2019	Residence	Oyam District	Number of ORS packages delivered (Hospital + Health Centers)	Total number of diarrhoea cases (<5 years)	14766	5916	2,50
IDPD12	Average number of ORS packages delivered per patient with diarrhoea (<5years)	2018	Residence	Oyam District	Number of ORS packages delivered (Hospital + Health Centers)	Total number of diarrhoea cases (<5 years)	14416	5546	2,60
IDPD12	Average number of ORS packages delivered per patient with diarrhoea (<5years)	2017	Residence	Oyam District	Number of ORS packages delivered (Hospital + Health Centers)	Total number of diarrhoea cases (<5 years)	19092	8956	2,13
IDPD13	Average number of Zinc Tablets doses delivered per patient with diarrhoea (<5years)	2019	Residence	Oyam District	Number of Zinc Tablets doses delivered (Hospital + Health Centers)	Total number of diarrhoea cases (<5 years)	14766	6857	2,15
IDPD13	Average number of Zinc Tablets doses delivered per patient with diarrhoea (<5years)	2018	Residence	Oyam District	Number of Zinc Tablets doses delivered (Hospital + Health Centers)	Total number of diarrhoea cases (<5 years)	14416	6408	2,25
IDPD13	Average number of Zinc Tablets doses delivered per patient with diarrhoea (<5years)	2017	Residence	Oyam District	Number of Zinc Tablets doses delivered (Hospital + Health Centers)	Total number of diarrhoea cases (<5 years)	19092	10903	1,75
IDPD14	Percentage of deaths with a diagnosis of gastroenteritis	2019	Hospital	Oyam District	Number of deaths diagnosed with gastroenteritis (patients aged < 5 years) (x10	Number of discharged patients with a diagnosis of gastroenteritis (patients aged < 5	0	104	0,00
IDPD14	Percentage of deaths with a diagnosis of gastroenteritis	2018	Hospital	Oyam District	Number of deaths diagnosed with gastroenteritis (patients aged < 5 years) (x10	Number of discharged patients with a diagnosis of gastroenteritis (patients aged < 5	2	205	0,98
IDPD14	Percentage of deaths with a diagnosis of gastroenteritis	2017	Hospital	Oyam District	Number of deaths diagnosed with gastroenteritis (patients aged < 5 years) (x10	Number of discharged patients with a diagnosis of gastroenteritis (patients aged < 5	0	360	0,00
IDPT01	Percentage of treatments with isoniazide (IPT)	2019	Residence	Oyam District	Number of treatments with isoniazide (IPT) (x100)	Total number of eligible treatments	94	367	25,61
IDPT01	Percentage of treatments with isoniazide (IPT)	2018	Residence	Oyam District	Number of treatments with isoniazide (IPT) (x100)	Total number of eligible treatments	70	431	16,24
IDPT01	Percentage of treatments with isoniazide (IPT)	2017	Residence	Oyam District	Number of treatments with isoniazide (IPT) (x100)	Total number of eligible treatments	2	155	1,29
IDPT02	Percentage of TB cases undergoing the HIV screening	2019	Residence	Oyam District	Number of TB cases undergoing the HIV screening (x100)	Number of TB diagnosed patients	561	594	94,44
IDPT02	Percentage of TB cases undergoing the HIV screening	2018	Residence	Oyam District	Number of TB cases undergoing the HIV screening (x100)	Number of TB diagnosed patients	445	468	95,09
IDPT02	Percentage of TB cases undergoing the HIV screening	2017	Residence	Oyam District	Number of TB cases undergoing the HIV screening (x100)	Number of TB diagnosed patients	311	319	97,49
IDPT05	Percentage of confirmed PTB	2019	Residence	Oyam District	Number of positive PTB cases (bacteriologically confirmed) (x100)	Number of PTB cases	#RIF!	576	#RIF!
IDPT05	Percentage of confirmed PTB	2018	Residence	Oyam District	Number of positive PTB cases (bacteriologically confirmed) (x100)	Number of PTB cases	#RIF!	446	#RIF!
IDPT05	Percentage of confirmed PTB	2017	Residence	Oyam District	Number of positive PTB cases (bacteriologically confirmed) (x100)	Number of PTB cases	#RIF!	286	#RIF!
IDPT06	Percentage of positive Xpert cases	2019	Hospital	Oyam District	Numer of positive Xpert cases (x100)	Number of Xpert cases	246	1558	15,79
IDPT06	Percentage of positive Xpert cases	2018	Hospital	Oyam District	Numer of positive Xpert cases (x100)	Number of Xpert cases	224	1239	18,08
IDPT06	Percentage of positive Xpert cases	2017	Hospital	Oyam District	Numer of positive Xpert cases (x100)	Number of Xpert cases	4	93	4,30
IDPT07	Percentage of treatments for extrapulmunary TB	2019	Residence	Oyam District	Number of treatments "initiated" for extrapolmunary TB (x100)	Number of TB diagnoses	25	594	4,21
IDPT07	Percentage of treatments for extrapulmunary TB	2018	Residence	Oyam District	Number of treatments "initiated" for extrapolmunary TB (x100)	Number of TB diagnoses	16	468	3,42
IDPT07	Percentage of treatments for extrapulmunary TB	2017	Residence	Oyam District	Number of treatments "initiated" for extrapolmunary TB (x100)	Number of TB diagnoses	13	293	4,44
IDPT09	Percentage of cured patients	2019	Residence	Oyam District	Number of cured patients (x100)	Number of PTB+ (bacteriologically confirmed)	190	502	37,85
IDPT09	Percentage of cured patients	2018	Residence	Oyam District	Number of cured patients (x100)	Number of PTB+ (bacteriologically confirmed)	191	379	50,40
IDPT09	Percentage of cured patients	2017	Residence	Oyam District	Number of cured patients (x100)	Number of PTB+ (bacteriologically confirmed)	85	261	32,57
IDPT10	Percentage of completed treatments	2019	Residence	Oyam District	Number of completed treatments (x100)	Number of treated cases	378	528	71,59
IDPT10	Percentage of completed treatments	2018	Residence	Oyam District	Number of completed treatments (x100)	Number of treated cases	415	564	73,58
IDPT10	Percentage of completed treatments	2017	Residence	Oyam District	Number of completed treatments (x100)	Number of treated cases	228	284	80,28
IDPT12	Percentage of interrupted treatments	2019	Residence	Oyam District	Number of interrupted treatments (x100)	Number of treated cases	78	528	14,77
IDPT12	Percentage of interrupted treatments	2018	Residence	Oyam District	Number of interrupted treatments (x100)	Number of treated cases	39	564	6,91
IDPT12	Percentage of interrupted treatments	2017	Residence	Oyam District	Number of interrupted treatments (x100)	Number of treated cases	26	284	9,15
