# Supplementary material for: An integrated hospital-district performance evaluation for communicable diseases in low-and middle-income countries: Evidence from a pilot in three sub-Saharan countries
Source: PLoS One. 2022 Mar 31;17(3):e0266225. doi: 10.1371/journal.pone.0266225 (PMC8970489; doi:10.1371/journal.pone.0266225)
Supplement: Attachment — Submitted filename: Response to Reviewers.docx [file pone.0266225.s005.docx]

**Review Comments to the Authors**

**Reviewer #1**

The paper is interesting and the approach to the evaluation of health services is innovative for Sub-Saharan Africa. However, one major general point and some minor ones should be addressed to make the manuscript valuable for pubblication.

| In general the paper seems to be more focused on the methods rather than the results (more evident in the abstract and in the discussion). If you intent is to present and innovative way to evaluate health services in Africa, you could consider another type of paper, focused on methodology. Otherwise you should focus more on the results. How the services you selected performed in the TB, HIV and gastroenteritis care?  In other words the logic of the research is not linear. The objective is to evaluate the tool (PES-CDs), but the results of the research are about health services. Maybe the objective was to evaluate the health services using an innovative tool? | Thank you for pointing out the necessity to provide a clear objective of the manuscript and an unambiguous linearity of our research flow. Following your suggestion, we decided to focus more on the performance results obtained by piloting the evaluation tool in the four selected settings rather than on the methodology that we followed to develop the system. Therefore, we changed the overall objective of the manuscript in the introduction section. Additionally, we revised the methods, results and discussion sections with the final aim of presenting and discussing: 1) the feasibility of implementing a PES-CD in LMICs and, 2) performance results derived from the use of the evaluation tool in the four selected settings. Therefore, all the sections of the paper, including the abstract, were revised accordingly. |
| --- | --- |

Minor comments:

Abstract:

| Consider structuring the abstract in sections, as you did in the main body of the paper. | We addressed your request. |
| --- | --- |
| Please, include results in the abstract | We addressed your request by including in the abstract the main findings deriving from the analyses of the three care pathways. |

Material and methods:

| is the NGO you mention CUAMM? You never state it clearly, why not? | Thank you for your suggestion. We explicitly mentioned Doctors with Africa CUAMM in the material and methods section (see rows 145 and 146). |
| --- | --- |

Results:

| Line 205-206: What do you mean by “correlate”? Do you have any measure of this correlation? If not maybe a more vague wording could be more indicated. | Thank you. The word “correlated” was inserted inappropriately. However, according to the new version of the results section, we decided to delete this specific part. |
| --- | --- |
| Line 216-219: This is more to be considered as a discussion point, not to be included in the results section. | We took into account your suggestion and we included this specific point in the discussion section (see rows 408 – 412). |

Discussion:

| Please, start the discussion with the main findings of the paper, then start discussing them.  Your discussion seems to be more focused on the method you used rathern than the results. However this is not a mothodological paper. The reccomandation is to rewrite the discussion starting from the main results in the performance of the services for the considered diseases and try to comapre them with similar data in the same or other contexts. | We addressed your request and, as abovementioned, we changed the discussion section according to new overall objective and flow of the manuscript. Therefore, in the discussion section, after having introduced the core features of the evaluation tool, we included the main findings deriving from the performance evaluation and benchmarking of the health services provision across all the contexts analysed.  Additionally, we agree with you that this is not a methodological paper. However, we believe the report of the development and piloting phases of the PES-CD represents an integral part of the manuscript. Therefore, we included in the discussion section a critical evaluation of challenges and opportunities that emerged during the development and piloting phase and concerns regarding feasibility to inform future research and project.  Finally, while discussing the findings, we focused on comparing the different settings analysed within the pilot study. This choice was grounded on the need to assess future transferability of the PES-CD across different settings in LMICs. |
| --- | --- |

**Reviewer #2**

 Some notes about the article:

| - the present study has great potential, uses several data, a consistent instrument, but needs greater adequacy in terms of structure for exposing data, results and even discussion; | - |
| --- | --- |
| - there is a certain inconsistency between title-objective-results, making the article lengthy to read, sometimes repetitive, however, not making it clear what is, in fact, the main objective of the study; | Thank you for pointing out this important issue. Following your suggestion, we revised the manuscript in all of its sections with the aim of reducing the inconsistency between title-objective-results and improving the overall research flow. |
| - OBJECTIVE/Title: in my view, objective and title are not integrated and articulated, in addition to the authors mentioning different objectives throughout the text, leaving the reader confused about what is intended to be explained; The authors mention at least 3 objectives: “evaluating the performance of the patient journey within the local healthcare system, thus assessing its capacity to provide healthcare services along the continuum of care and through the integrations of different organizations”; “we aimed at describing the development of a PES of health services provision related to CDs (PES-CDs) in three selected sub-Saharan countries”, AND “evaluating the performance of the local healthcare system along the patient continuum care”; | Thank you for your comment. We stated more explicitly and clearly the main objective of the paper both in the abstract and introduction sections, deleting repetitive and confounding concepts along the wording of the manuscript.  More particularly, we decided to revise the manuscript’s title and overall objective, that is now aimed at presenting and discussing: 1) the feasibility of implementing a PES-CD in LMICs and, 2) performance results derived from the use of the evaluation tool in the four selected settings. Therefore, all the sections of the paper, including the abstract, were revised accordingly. |
| - the authors use the term “pathways” and “journey” giving the impression to the reader that they will deal with the paths of the patient through the health system, but in fact, I understand that these are care actions against three communicable diseases; | Thank you for your comment, we agree that the use of the tem “journey” may not be appropriate and we deleted it from the manuscript.  However, we maintained the term pathway to refer to the “care pathway” specific to each communicable disease studied. We developed a set of indicators which take into consideration the different (and subsequent) care phases, namely prevention (or screening), diagnosis, treatment and outcome, representing the pathway of care for that specific disease. |
| - at other times, the reader has the impression that the main objective of the study, in fact, is to validate an instrument for performance evaluation; | We agree with you. As abovementioned, the focus is not on the validation of the performance evaluation tool, but on the discussion of the performance results derived from the use and implementation of the evaluation system in the four selected settings. |
| - the article has a lot of data to be explored and it might be prudent for the authors to organize a better title, objective, methods, results and discussion, so that this can be made clear to the reader; | Thank you for your input, please see the above comment. We addressed your suggestions. |
| - the present study has very robust data and is extremely important for the scientific community, as well as for the countries covered, however, it is necessary to carry out a broad review about the organization of the data and what, in fact, is intended respond with the results obtained. | Following your suggestion, we decided to focus more on the performance results obtained by piloting the evaluation tool in the four selected settings rather than on the methodology that we followed to develop the system. We addressed your input and we changed the discussion section according to new overall objective and flow of the manuscript. Therefore, in the discussion section, after having introduced the core features of the evaluation tool, we included the main findings deriving from the performance evaluation and benchmarking of the health services provision across all the contexts analysed. While this is not intended to be a methodological paper, we believe the report of the development and piloting phase of the PES is an integral part of the manuscript. Therefore, we included in the discussion a critical evaluation of challenges that emerged during the development and piloting phases as well as concerns regarding feasibility and transferability of the PES-CD across different settings in LMICs. |
